# Patient satisfaction with dialysis services provided across different providers in Saudi Arabia

**DOI:** 10.3389/fneph.2025.1691773

**Published:** 2026-01-12

**Authors:** Sarah S. Monshi, Hatoon M. Alamri, Afnan M. Almuaddi, Fatemah M. Almutairi, Hala R. Aljishi, Khulud A. Alfaki, Maram S. AlTurki, Rayyan M. Saqah, Mohammed S. Aldossary

**Affiliations:** 1 Department of Health Administration and Hospitals, College of Public Health and Health Informatics, Umm Al-Qura University, Makkah, Saudi Arabia; 2 General Directorate of Research and Studies, Ministry of Health, Riyadh, Saudi Arabia; 3 Laboratory and Blood Bank Department, King Abdullah Medical City, Makkah, Saudi Arabia; 4 General Administration of School Health, Ministry of Health, Riyadh, Saudi Arabia; 5 Global Center for Mass Gathering Medicine, Ministry of Health, Riyadh, Saudi Arabia; 6 Minister’s Office, International Health Indicators Unit, Ministry of Health, Riyadh, Saudi Arabia; 7 Evaluation and Impact Measurement Unit, Public Health Operations Center (PHOC), Ministry of Health, Riyadh, Saudi Arabia; 8 General Directorate of Environmental Health, Ministry of Health, Riyadh, Saudi Arabia

**Keywords:** dialysis satisfaction, healthcare facilities, healthcare quality, hemodialysis, patient experience, Saudi Arabia

## Abstract

**Objectives:**

This study aimed to evaluate patient satisfaction with dialysis services provided across different healthcare sectors in Saudi Arabia, including governmental and private facilities, and to identify key determinants influencing satisfaction levels.

**Methods:**

A cross-sectional observational study was conducted using secondary data from dialysis patients attending Ministry of Health, Diaverum, and DaVita facilities between January and December 2023. Patient satisfaction data were collected through the Press Ganey survey, a validated instrument assessing six domains: registration, care, dialysis, pharmacy, personal issues, and personal experience. Descriptive statistics summarized patient demographics and satisfaction scores, while regression analysis identified factors associated with satisfaction.

**Results:**

A total of 5,472 patients were included, with an overall satisfaction score of 89.84 ± 14.25. The mean satisfaction score was highest in the personal experience domain (91.39 ± 17.02) and lowest in the dialysis domain (88.45 ± 18.65). Private facilities had statistically significant higher satisfaction scores (90.41 ± 13.31) compared to governmental hospitals (88.57 ± 16.08). Females reported significantly higher satisfaction than males (91.96 ± 12.15 vs. 88.91 ± 14.60), respectively. Pediatric patients demonstrated significantly higher satisfaction (age ≤18 years: 93.80 ± 11.42) compared to young adults (age = 19–29 years: 89.18 ± 14.62). Regional differences were observed, with the Southern region reporting the highest satisfaction (91.37 ± 14.18) and the Eastern region the lowest (88.60 ± 15.59). Regression analysis identified gender (B = 2.943, 95% CI [2.165, 3.722], p < 0.001) and facility type (B = 1.108, 95% CI [0.243, 1.973], p = 0.012) as significant predictors of satisfaction.

**Conclusion:**

Patient satisfaction with dialysis services in Saudi Arabia is generally high, with statistically significant but modest differences across regions, facility types, age groups, and genders. Improving dialysis-related education, addressing regional disparities, and enhancing patient-centered care, particularly in governmental facilities, could further optimize satisfaction outcomes.

## 1 Introduction

End-stage renal disease (ESRD) presents a significant and growing health challenge in Saudi Arabia (1), primarily due to the rising prevalence of chronic conditions such as diabetes and hypertension (2). These diseases are leading contributors to ESRD, as they progressively damage kidney function over time. As of 2021, over 20,000 patients were undergoing dialysis, with an additional 9,810 individuals receiving post-kidney transplant follow-up, resulting in a combined renal replacement therapy prevalence of approximately 294.3 per million people (3, 4). The introduction of dialysis services in the early 1970s marked a pivotal advancement in Saudi Arabia’s healthcare system (5). Since then, the number of dialysis centers has expanded substantially, reaching 275 facilities equipped with over 8,000 hemodialysis machines by 2021. The distribution of these centers varies across the country’s regions, with the central and western areas hosting the majority of facilities (6).

Despite technological advancements and the expansion of services, the increasing prevalence of ESRD continues to impose substantial burdens on patients, healthcare systems, and the country. Patients endure rigorous treatment regimens and frequent hospital visits, often leading to a diminished quality of life (7). Healthcare systems face escalating demands for dialysis services, straining resources and infrastructure. Economically, the impact is profound, with significant portions of healthcare budgets allocated to managing ESRD and its associated complications (8). This highlights the critical importance of implementing robust healthcare quality assurance programs and prioritizing patient satisfaction.

A recent cross-sectional study conducted in the Ha’il region of Saudi Arabia assessed the quality of life (QoL) and needs of 222 hemodialysis patients. The findings revealed that approximately two-thirds of participants reported good QoL and satisfaction with their health. The study also highlighted that older patients (over 60 years) and those with higher BMI reported lower QoL and greater needs of specialized care (9). Another cross-sectional study involving 322 hemodialysis patients across three Saudi cities, Riyadh, Dammam, and Buraidah, revealed notable disparities in patient satisfaction levels. The overall mean satisfaction score was highest in Riyadh, followed by Dammam and Buraidah. Factors contributing to lower satisfaction included male gender, extended duration on dialysis (>3 years), and lower educational attainment (<4 years). Specifically, patients with less education reported greater negative impacts of dialysis on stress, overall health, sexual life, hobbies, and physical activity (10). Findings from both studies indicate that disadvantaged populations, such as the elderly, people with obesity, and those with lower education levels and longer dialysis durations, experience more significant challenges, highlighting the need for targeted interventions to enhance their dialysis experience and overall quality of life.

The Saudi government has proactively addressed these challenges through initiatives such as the National Transformation Programs within the Saudi Vision 2030, aiming to enhance healthcare infrastructure and service quality (11). These programs emphasize the importance of quality assurance and patient satisfaction as integral components of healthcare delivery. Patient satisfaction is recognized as a critical indicator of healthcare quality, influencing treatment adherence, continuity of care, and overall health outcomes (12). Studies have demonstrated that satisfied patients are more likely to engage positively with healthcare systems, leading to improved compliance and better clinical results (13, 14).

Given the lack of sufficient evidence regarding patients’ satisfaction with the hemodialysis service provided in Saudi Arabia, this study aims to evaluate patient satisfaction with dialysis services offered by the public sector “Ministry of Health (MOH)” and other private providers. The study provides insights for healthcare policymakers and administrators to enhance service delivery and patient care nationwide.

## 2 Materials and methods

### 2.1 Study design

This cross-sectional observational study utilized secondary data from patients attending private international dialysis facilities, including Diaverum and DaVita, as well as MOH dialysis facilities across the Kingdom of Saudi Arabia between January and December 2023. During this period, the national MOH patient experience program was active across MOH-operated and MOH-contracted dialysis centers, and invitations to complete the patient experience survey were routinely generated for patients with registered mobile phone numbers following their dialysis sessions. Data were sourced from the MOH’s patient experience program, which engaged Press Ganey, an independent third-party renowned for evaluating and enhancing patient experiences globally. The involvement of Press Ganey aimed to enhance data quality by minimizing potential biases in data collection and analysis. The administered questionnaire comprised non-mandatory items, allowing patients the discretion to provide complete or partial responses. The study was conducted in accordance with ethical principles governing research involving human subjects. Ethical approval for this study was obtained from the Central Institutional Review Board of the Saudi MOH (IRB log No: 25-37M). Participants’ informed consent for research use was obtained prior to data collection. Confidentiality and anonymity of participant information were rigorously maintained throughout the research process, with data securely stored on MOH servers and access restricted to authorized personnel.

### 2.2 Setting and participants

The study included patients who received dialysis services at Diaverum, DaVita, and MOH facilities throughout Saudi Arabia during the specified period and who completed the patient experience survey. Eligible participants were at all ages, with survey participation considered as implied consent to the patient experience satisfaction questionnaire.

### 2.3 Data collection

Data were collected via the Press Ganey survey questionnaire, a self-administered instrument designed to assess patient experience and satisfaction (**Supplementary File A**). The Press Ganey survey is a widely validated instrument used in over 35,000 healthcare facilities worldwide (15–17). In Saudi Arabia, the dialysis version was developed for the national Patient Experience Measurement Program and its Arabic version was produced in accordance with the World Health Organization’s guidelines for the translation and adaptation of instruments, including forward–back translation, expert review, and pilot testing (18). A concise summary of the instrument’s domains, response options, scoring approach, and available psychometric evidence is provided in **Supplementary File B**.

The questionnaire was disseminated to patients’ registered mobile phones following each dialysis session. To ensure data integrity, responses with more than 50% missing data were excluded from the analysis. The questionnaire employed a 5-point Likert scale, ranging from 1 (“not satisfied at all”) to 5 (“extremely satisfied”), to gauge satisfaction levels across various service dimensions. Item responses within each domain were aggregated and reported as domain scores on a 0–100 scale, with higher values indicating greater satisfaction. An overall satisfaction score was similarly derived from the four items in the overall assessment domain (e.g. overall rating of care and likelihood of recommending the center) and expressed on the same 0–100 scale. It comprised 22 items categorized into six domains pertinent to dialysis care: registration (4 items), care (6 items), dialysis process (3 items), pharmacy services (3 items), personal interactions (2 items), and overall assessment (4 items). The survey was available in both Arabic and English and was typically sent to patients or their guardians via registered mobile numbers shortly after their visit to the dialysis unit.

### 2.4 Data analysis

Descriptive statistics, including percentages, means, medians, and standard deviations, were conducted to summarize patient demographics and satisfaction scores. Comparisons between two independent groups were performed using Student’s t-tests, while analyses of variance (ANOVA) assessed differences among multiple groups. A p-value of less than 0.05 was considered indicative of statistical significance. Pearson correlation coefficients were calculated to evaluate the strength and direction of linear relationships between variables. Subsequently, regression analyses were performed to identify factors that were significantly associated with patient satisfaction levels. No anchor-based minimal clinically important difference (MCID) has been established for the Press Ganey dialysis survey or for the 0–100 satisfaction domain scores used in this study, and our dataset did not contain an external anchor (e.g., global rating of change) that would allow us to derive one empirically. Therefore, we interpreted between-group differences using a distribution-based framework rather than a formal anchor-based MCID. Consistent with commonly used conventions for patient-reported outcomes, we considered differences of approximately 0.5 standard deviations (SD) in overall satisfaction (approximately 7 points on the 0–100 scale in our sample) to represent a moderate, potentially clinically important difference, whereas smaller differences (around 0.2 SD, around 3 points) were regarded as small effects (19). Statistical analyses were conducted using the Statistical Package for the Social Sciences (SPSS) software (IBM Inc., Chicago, Version 21.0).

## 3 Results

### 3.1 Demographic characteristics

A total of 5,580 patients responded to the survey, of which 5,472 (98.1%) were fully completed and included in the analysis. Participants with incomplete responses (1.9%) were excluded from the analysis. The mean age and standard deviation of participants was 51.97 ± 15.49 years. Males accounted for 61.1% of the study sample, while females comprised 38.9% of the participants. Regarding facility type, 68.6% of the participants received care in private facilities, while 31.4% were treated in MOH facilities. Among private centers, 53.4% of patients were in DaVita clinics and 46.6% in Diaverum clinics. Regional distribution data showed that the highest proportion of participants from the Western region (30.4%), followed by the Central (24.6%), Southern (23.0%), Eastern (13.5%), and Northern (8.5%) regions (**Table 1**).

**Table 1:** Demographic characteristics of patients receiving dialysis services.

Variables		Total (N = 5472)
Age, years	n/missing	5076/396
	Mean (SD)	51.97 (15.49)
	Median	53.00
	≤18	56 (1.1%)
	19-29	364 (7.2%)
	30-64	3538 (69.7%)
	≥65	1118 (22.0%)
Gender, n (%)	n/missing	5072/401
	Male	3099 (61.1%)
	Female	1972 (38.9%)
Facility Type, n (%)	n/missing	5461/11
	MOH	1717 (31.4%)
	Private	3744 (68.6%)
Private Facilities, n (%)	n/missing	3744/0
	DaVita	2000 (53.4%)
	Diaverum	1744 (46.6%)
Region, n (%)	n/missing	5461/11
	Central	1341 (24.6%)
	Eastern	735 (13.5%)
	Northern	465 (8.5%)
	Western	1662 (30.4%)
	Southern	1258 (23.0%)

### 3.2 Patient satisfaction with dialysis services

The overall satisfaction score among participants was 89.84 ± 14.25, as shown in **Table 2**. In the registration domain, participants reported a mean satisfaction score of 88.82 ± 16.22, with 72.5% expressing extreme satisfaction regarding the helpfulness of the receptionist and 71.8% for the ease of the registration process. However, satisfaction with the waiting time in registration was slightly lower, with 66.4% being extremely satisfied. Regarding the care domain, the mean satisfaction score was 90.56 ± 15.66. Notably, 82.7% of participants felt they were treated with respect and dignity, while 75.7% were extremely satisfied with the staff’s concern for their comfort and the opportunity to ask questions. Trust in the medical staff’s skills was also high, with 71.8% expressing extreme satisfaction. In contrast, responses to concerns and complaints showed a lower level of satisfaction, with 61.7% reporting being extremely satisfied. In the dialysis domain, the mean score was 88.45 ± 18.65, with 76.7% extremely satisfied with staff monitoring their dialysis care, while instructions on managing dialysis complications at home had a slightly lower satisfaction rate of 61.6%. The pharmacy domain had a mean satisfaction score of 89.50 ± 17.34, with 77.1% extremely satisfied with pharmacist explanations, but the availability of prescribed medications received lower ratings, with 65.8% reporting extreme satisfaction. The personal issues domain received a mean score of 90.98 ± 16.41, with 78.1% extremely satisfied with privacy concerns and 72.7% with facility cleanliness. The personal experience domain had the highest satisfaction, with a mean score of 91.39 ± 17.02. Staff teamwork in providing care was rated extremely satisfactory by 78.5% of participants, while 74.6% expressed extreme satisfaction with their likelihood of recommending the center.

**Table 2:** Patient satisfaction across dialysis service domains in Saudi Arabia.

Domains	N/missing	Not satisfied at all	Not satisfied	Moderate	Satisfied	Extremely satisfied	Mean (SD)	95% CI
		n (%)	n (%)	n (%)	n (%)	n (%)		
Registration	5446/26						88.82 (16.22)	(88.39, 89.25)
Helpfulness of the receptionist	5425/47	56 (1.0%)	20 (0.4%)	154 (2.8%)	1261 (23.2%)	3934 (72.5%)		
Ease of the registration process.	5434/38	63 (1.2%)	23 (0.4%)	176 (3.2%)	1273 (23.4%)	3899 (71.8%)		
Waiting time in registration.	5238/234	123 (2.3%)	67 (1.3%)	243 (4.6%)	1328 (25.4%)	3477 (66.4%)		
Comfort of the waiting area	5028/444	253 (5.0%)	68 (1.4%)	311 (6.2%)	1321 (26.3%)	3075 (61.2%)		
Care	5471/1						90.56 (15.66)	(90.14, 90.97)
Medical staff’s explanation of the dialysis procedure	5457/15	104 (1.9%)	43 (0.8%)	226 (4.1%)	951 (17.4%)	4133 (75.7%)		
Medical staff provided opportunity to ask questions	5439/33	97 (1.8%)	51 (0.9%)	182 (3.3%)	1022 (18.8%)	4087 (75.1%)		
Your trust in the skill of the medical staff	5467/5	89 (1.6%)	42 (0.8%)	251 (4.6%)	1159 (21.2%)	3926 (71.8%)		
Staff’s concern for your comfort	5469/3	87 (1.6%)	41 (0.7%)	191 (3.5%)	1008 (18.4%)	4142 (75.7%)		
Staff treated you with respect and dignity	5470/2	55 (1.0%)	20 (0.4%)	127 (2.3%)	746 (13.6%)	4522 (82.7%)		
Response to concerns/complaints made during your visit	4315/1157	263 (6.1%)	129 (3.0%)	267 (6.2%)	994 (23.0%)	2662 (61.7%)		
Dialysis	5433/39						88.45 (18.65)	(87.95, 88.94)
Staff’s monitoring of your dialysis care	5462/10	80 (1.5%)	42 (0.8%)	180 (3.3%)	973 (17.8%)	4187 (76.7%)		
Staff’s instructions on how to care for your dialysis access	5111/361	116 (2.3%)	61 (1.2%)	210 (4.1%)	999 (19.5%)	3725 (72.9%)		
Instructions about what to do in case of any complications related to dialysis at home	5309/163	265 (5.0%)	130 (2.4%)	351 (6.6%)	1294 (24.4%)	3269 (61.6%)		
Pharmacy	4580/892						89.50 (17.34)	(89.00, 90.00)
Explanations provided by pharmacist about prescription(s)	4522/950	45 (1.0%)	24 (0.5%)	132 (2.9%)	833 (18.4%)	3488 (77.1%)		
Availability of prescribed medications	4575/897	123 (2.7%)	102 (2.2%)	298 (6.5%)	1043 (22.8%)	3009 (65.8%)		
Personal Issues	5448/24						90.98 (16.41)	(90.54, 91.41)
Our concern for your privacy	5441/31	71 (1.3%)	39 (0.7%)	158 (2.9%)	921 (16.9%)	4252 (78.1%)		
Cleanliness of the facility	5444/28	147 (2.7%)	46 (0.8%)	274 (5.0%)	1017 (18.7%)	3960 (72.7%)		
Personal Experience	5443/29						91.39 (17.02)	(90.94, 91.84)
How well staff worked together to provide care	5441/31	64 (1.2%)	29 (0.5%)	174 (3.2%)	905 (16.6%)	4269 (78.5%)		
Likelihood of your recommending our center to others	5417/55	128 (2.4%)	69 (1.3%)	237 (4.4%)	944 (17.4%)	4039 (74.6%)		
Overall Satisfaction	5472/0						89.84 (14.25)	(89.46, 90.22)

### 3.3 Patient satisfaction score by region

The Southern region had a statistically significantly higher overall satisfaction score (91.37 ± 14.18) compared to the Central region (90.10 ± 13.48), with a mean difference (MD) of 1.28 (95% CI: 0.18–2.37; p=0.022). The Eastern region had the lowest overall satisfaction score (88.60 ± 15.59), which was statistically significantly lower than the Central region by 1.49 points (95% CI: -2.77 to -0.21; p=0.022), as shown in **Table 3** and **Supplementary File C/Table 1**. However, the absolute differences in mean scores between regions were small, indicating that these regional contrasts are statistically significant but unlikely to be clinically important. This trend was consistent across most domains, with the Southern region consistently showing the highest satisfaction, particularly in care (92.15 ± 15.51), dialysis (90.77 ± 17.5), personal issues (92.28 ± 16.03), and personal experience (92.49 ± 16.84). The Eastern region had the lowest satisfaction scores in care (88.62 ± 17.77), dialysis (86.75 ± 20.07), and overall satisfaction (88.6 ± 15.59). The results of the *post-hoc* analysis comparing satisfaction scores across regions are presented in **Supplementary File C/Table 2**.

**Table 3:** The mean of patient satisfaction score categorized by region.

Region	Domain	n/missing	Mean (SD)	95% CI
Central	Registration	1330/11	88.78 (15.82)	(87.93, 89.63)
	Care	1341/0	90.9 (14.74)	(90.11, 91.69)
	Dialysis	1325/16	88.35 (18.15)	(87.37, 89.33)
	Pharmacy	1069/272	89.45 (16.93)	(88.44, 90.47)
	Personal Issues	1335/6	91.77 (14.96)	(90.97, 92.57)
	Personal Experience	1335/6	91.67 (16.49)	(90.78, 92.55)
	Overall Satisfaction	1341/0	90.1 (13.48)	(89.37, 90.82)
Eastern	Registration	732/3	87.6 (17.79)	(86.31, 88.89)
	Care	735/0	88.62 (17.77)	(87.33, 89.91)
	Dialysis	732/3	86.75 (20.07)	(85.3, 88.21)
	Pharmacy	628/107	89.33 (17.53)	(87.96, 90.7)
	Personal Issues	731/4	90.13 (17.78)	(88.84, 91.42)
	Personal Experience	729/6	90.28 (19)	(88.9, 91.66)
	Overall Satisfaction	735/0	88.6 (15.59)	(87.48, 89.73)
Northern	Registration	462/3	88.82 (16.19)	(87.34, 90.3)
	Care	465/0	90.23 (15.17)	(88.85, 91.61)
	Dialysis	463/2	87.35 (20.41)	(85.48, 89.21)
	Pharmacy	375/90	88.1 (19.91)	(86.08, 90.12)
	Personal Issues	461/4	90.32 (17.06)	(88.76, 91.88)
	Personal Experience	460/5	91.06 (16.35)	(89.56, 92.56)
	Overall Satisfaction	465/0	89.13 (15.08)	(87.75, 90.5)
Western	Registration	1656/6	88.37 (15.45)	(87.63, 89.12)
	Care	1661/1	90 (15.56)	(89.25, 90.75)
	Dialysis	1650/12	87.78 (18.64)	(86.88, 88.68)
	Pharmacy	1431/231	88.99 (17.24)	(88.1, 89.89)
	Personal Issues	1658/4	89.87 (16.93)	(89.05, 90.68)
	Personal Experience	1657/5	90.89 (16.84)	(90.08, 91.7)
	Overall Satisfaction	1662/0	89.18 (13.99)	(88.51, 89.85)
Southern	Registration	1255/3	90.15 (16.62)	(89.23, 91.07)
	Care	1258/0	92.15 (15.51)	(91.29, 93.01)
	Dialysis	1252/6	90.77 (17.5)	(89.8, 91.74)
	Pharmacy	1066/192	90.75 (16.8)	(89.74, 91.76)
	Personal Issues	1252/6	92.28 (16.03)	(91.39, 93.17)
	Personal Experience	1251/7	92.49 (16.84)	(91.55, 93.42)
	Overall Satisfaction	1258/0	91.37 (14.18)	(90.59, 92.16)

### 3.4 Patient satisfaction score by facility type

Overall, private hospitals reported a significantly higher satisfaction score (90.41 ± 13.31) compared to governmental hospitals (88.57 ± 16.08), with an MD of 1.83 (95% CI: 1.02-2.65; p<0.001), as reported in **Supplementary File C/Table 1**. Nonetheless, this statistically significant difference in overall satisfaction between private and governmental facilities was mild in magnitude and is unlikely to reflect a major difference in patients’ perceived quality of care. This trend was consistent across all domains, with private hospitals scoring higher in registration (89.01 ± 15.71 vs. 88.38 ± 17.28), care (90.79 ± 15.07 vs. 90.04 ± 16.89), dialysis (88.89 ± 17.65 vs. 87.43 ± 20.7), personal issues (91.4 ± 15.79 vs. 90.00 ± 17.68), and personal experience (92.05 ± 16.04 vs. 89.91 ± 18.95) domains. The pharmacy domain showed the largest difference, with private hospitals scoring significantly higher (91.04 ± 15.36) than governmental hospitals (85.79 ± 20.88), as shown in **Table 4**.

**Table 4:** Satisfaction score categorized by facility type and type of private center.

Facility type	Domain	N/missing	Mean (SD)	95% CI
Governmental	Registration	1697/20	88.38 (17.28)	(87.55, 89.2)
	Care	1716/1	90.04 (16.89)	(89.24, 90.84)
	Dialysis	1697/20	87.43 (20.7)	(86.44, 88.41)
	Pharmacy	1356/361	85.79 (20.88)	(84.68, 86.91)
	Personal Issues	1707/10	90 (17.68)	(89.17, 90.84)
	Personal Experience	1704/13	89.91 (18.95)	(89.01, 90.81)
	Overall Satisfaction	1717/0	88.57 (16.08)	(87.81, 89.33)
Private	Registration	3738/6	89.01 (15.71)	(88.51, 89.52)
	Care	3744/0	90.79 (15.07)	(90.3, 91.27)
	Dialysis	3725/19	88.89 (17.65)	(88.32, 89.46)
	Pharmacy	3213/531	91.04 (15.36)	(90.51, 91.57)
	Personal Issues	3730/14	91.4 (15.79)	(90.9, 91.91)
	Personal Experience	3728/16	92.05 (16.04)	(91.54, 92.57)
	Overall Satisfaction	3744/0	90.41 (13.31)	(89.98, 90.83)
DaVita	Registration	1998/2	89.18 (15.86)	(88.49, 89.88)
	Care	2000/0	90.2 (15.86)	(89.5, 90.89)
	Dialysis	1989/11	88.5 (18.35)	(87.69, 89.31)
	Pharmacy	1724/276	91.14 (15.34)	(90.42, 91.86)
	Personal Issues	1992/8	90.96 (16.34)	(90.25, 91.68)
	Personal Experience	1990/10	91.73 (16.62)	(91, 92.46)
	Overall Satisfaction	2000/0	90.15 (13.8)	(89.54, 90.75)
Diaverum	Registration	1740/4	88.82 (15.55)	(88.09, 89.55)
	Care	1744/0	91.46 (14.09)	(90.8, 92.13)
	Dialysis	1736/8	89.34 (16.8)	(88.55, 90.13)
	Pharmacy	1489/255	90.93 (15.39)	(90.14, 91.71)
	Personal Issues	1738/6	91.91 (15.13)	(91.2, 92.62)
	Personal Experience	1738/6	92.42 (15.35)	(91.7, 93.14)
	Overall Satisfaction	1744/0	90.7 (12.71)	(90.11, 91.3)

### 3.5 Patient satisfaction score by private center

Overall, Diaverum reported a slightly higher satisfaction score (90.7 ± 12.71) compared to DaVita (90.15 ± 13.8), with no statistically significant difference (p=0.203), as shown in **Supplementary File C/Table 1**. This trend was observed across multiple domains, with satisfaction levels being similar in registration (89.18 ± 15.86 for DaVita vs. 88.82 ± 15.55 for Diaverum) and pharmacy (91.14 ± 15.34 for DaVita vs. 90.93 ± 15.39 for Diaverum). However, Diaverum showed higher satisfaction in care (91.46 ± 14.09 vs. 90.2 ± 15.86), dialysis (89.34 ± 16.8 vs. 88.5 ± 18.35), personal issues (91.91 ± 15.13 vs. 90.96 ± 16.34), and personal experience (92.42 ± 15.35 vs. 91.73 ± 16.62). Both centers demonstrated high satisfaction across all domains and the numerical differences between the two private providers were small and neither statistically nor clinically significant, as shown in **Table 4**.

### 3.6 Patient satisfaction score by participants’ age

Compared with patients aged ≤18 years (93.80 ± 11.42), those aged 19–29 years had a significantly lower overall satisfaction score (89.18 ± 14.62), with an MD of -4.62 (95% CI: -8.54 to -0.70; p=0.021). The 30–64 group (89.90 ± 14.1) also had significantly lower ratings than the ≤18 group, with an MD of -3.90 (95% CI: -7.58 to -0.23; p=0.037), as reported in **Supplementary File C/Table 1**. This trend was consistent across all domains, with the ≤18 group consistently showing the highest satisfaction scores, particularly in care (95.24 ± 11.11), dialysis (93.26 ± 14.8), personal issues (95.68 ± 11.59), and personal experience (95.45 ± 11.87). In contrast, the 19–29 group reported the lowest satisfaction levels in most domains, including dialysis (87.86 ± 19.41) and personal issues (89.39 ± 17.76). The 30–64 and ≥65 groups had relatively similar satisfaction scores, with slightly higher ratings in the care and personal experience domains for the ≥65 group (91.38 ± 14.26 and 92.44 ± 14.67, respectively), as shown in **Table 5**. These age-related differences in satisfaction were statistically significant, but the absolute gaps in mean scores were small to moderate, and their clinical relevance is likely limited. The results of the *post-hoc* analysis comparing satisfaction scores across age groups are presented in **Supplementary File C/Table 3**.

**Table 5:** Satisfaction score categorized by participants’ age and gender.

Group	Domain	n/missing	Mean (SD)	95% CI
≤18	Registration	56/0	91.67 (12.32)	(88.37, 94.96)
	Care	56/0	95.24 (11.11)	(92.26, 98.21)
	Dialysis	55/1	93.26 (14.8)	(89.26, 97.26)
	Pharmacy	46/10	92.93 (15.73)	(88.26, 97.61)
	Personal Issues	55/1	95.68 (11.59)	(92.55, 98.82)
	Personal Experience	55/1	95.45 (11.87)	(92.24, 98.66)
	Overall Satisfaction	56/0	93.8 (11.42)	(90.74, 96.86)
19-29	Registration	364/0	88.87 (15.98)	(87.22, 90.51)
	Care	364/0	89.65 (16.58)	(87.94, 91.36)
	Dialysis	362/2	87.86 (19.41)	(85.85, 89.86)
	Pharmacy	302/62	89.98 (17.97)	(87.95, 92.02)
	Personal Issues	363/1	89.39 (17.76)	(87.56, 91.23)
	Personal Experience	363/1	90.43 (17.84)	(88.59, 92.27)
	Overall Satisfaction	364/0	89.18 (14.62)	(87.67, 90.69)
30-64	Registration	3524/14	88.75 (16.28)	(88.21, 89.28)
	Care	3537/1	90.51 (15.69)	(89.99, 91.03)
	Dialysis	3514/24	88.49 (18.5)	(87.88, 89.11)
	Pharmacy	3042/496	90.09 (16.67)	(89.5, 90.69)
	Personal Issues	3526/12	90.92 (16.44)	(90.38, 91.46)
	Personal Experience	3525/13	91.39 (17.25)	(90.82, 91.96)
	Overall Satisfaction	3538/0	89.9 (14.1)	(89.43, 90.36)
≥65	Registration	1112/6	89.71 (14.57)	(88.85, 90.57)
	Care	1118/0	91.38 (14.26)	(90.54, 92.22)
	Dialysis	1111/7	88.87 (17.47)	(87.84, 89.9)
	Pharmacy	879/239	88.74 (17.21)	(87.6, 89.88)
	Personal Issues	1109/9	91.91 (14.77)	(91.04, 92.78)
	Personal Experience	1105/13	92.44 (14.67)	(91.58, 93.31)
	Overall Satisfaction	1118/0	90.47 (13.21)	(89.7, 91.25)
Male	Registration	3089/10	87.72 (16.81)	(87.12, 88.31)
	Care	3098/1	89.46 (16.49)	(88.88, 90.04)
	Dialysis	3080/19	87.01 (19.54)	(86.32, 87.7)
	Pharmacy	2590/509	89.03 (16.99)	(88.37, 89.68)
	Personal Issues	3087/12	90.57 (16.43)	(89.99, 91.15)
	Personal Experience	3085/14	90.51 (17.81)	(89.88, 91.14)
	Overall Satisfaction	3099/0	88.91 (14.6)	(88.39, 89.42)
Female	Registration	1963/9	91.13 (13.71)	(90.53, 91.74)
	Care	1972/0	92.8 (12.95)	(92.23, 93.37)
	Dialysis	1957/15	91.3 (15.4)	(90.62, 91.99)
	Pharmacy	1676/296	91.48 (16)	(90.72, 92.25)
	Personal Issues	1962/10	92.01 (15.46)	(91.33, 92.7)
	Personal Experience	1960/12	93.42 (14.32)	(92.78, 94.05)
	Overall Satisfaction	1972/0	91.96 (12.15)	(91.42, 92.49)

### 3.7 Patient satisfaction score by gender

Females reported a significantly higher overall satisfaction score (91.96 ± 12.15) compared to males (88.91 ± 14.6), with an MD of 3.05 (95% CI: 2.31-3.79; p<0.001), as shown in **Supplementary File C/Table 1**. Although statistically significant, this gender difference in overall satisfaction was small and should be interpreted as having limited clinical impact. A similar trend was observed across all satisfaction domains, with females consistently expressing greater satisfaction than males. The greatest differences were observed in the care (92.8 ± 12.95 vs. 89.46 ± 16.49), dialysis (91.3 ± 15.4 vs. 87.01 ± 19.54), and personal experience (93.42 ± 14.32 vs. 90.51 ± 17.81) domains. Similarly, satisfaction with registration was higher among females (91.13 ± 13.71) compared to males (87.72 ± 16.81), as well as in the pharmacy (91.48 ± 16.0 vs. 89.03 ± 16.99) and personal issues (92.01 ± 15.46 vs. 90.57 ± 16.43) domains (**Table 5**).

### 3.8 Correlation analysis of overall satisfaction and domain scores

The correlation analysis demonstrated uniformly strong positive associations between overall satisfaction and each domain score, with correlation coefficients ranging from 0.721 to 0.905 (all p<0.001). Patients who reported higher satisfaction within specific domains tended to report higher overall satisfaction with dialysis care. The care and personal experience domains had the numerically largest correlations with overall satisfaction (r = 0.905 and 0.885, respectively), whereas the pharmacy domain had the lowest (r = 0.721); however, given that all correlations were high, these differences are best interpreted as indicating that multiple aspects of care contribute jointly to overall satisfaction rather than identifying a single domain as uniquely dominant. At the item level, questions within the care and pharmacy domains also showed strong correlations with overall satisfaction (generally r>0.75), as shown in **Supplementary File C/Table 4**.

### 3.9 Regression analysis of overall satisfaction scores

Gender had the strongest association with satisfaction, with females exhibiting a significantly higher satisfaction score compared to males (β = 2.943, 95% CI: 2.165–3.722, p<0.001). Facility type was also a significant predictor, with private facilities associated with higher satisfaction levels compared to governmental facilities (β = 1.108, 95% CI: 0.243–1.973, p=0.012), as shown in **Table 6**.

**Table 6:** Regression analysis of overall satisfaction scores by facility types, age, and gender.

Parameter	B coefficient	95.0% confidence interval for B		P-value
		Lower bound	Upper bound	
Age	0.016	-0.008	0.041	0.192
Gender	2.943	2.165	3.722	<0.001
Facility Type	1.108	0.243	1.973	0.012

### 3.10 Effect of demographics on overall satisfaction

The subgroup analysis revealed significant differences in overall satisfaction across age groups, gender, and regions. Within the Central region, a significant difference was observed between age groups (p<0.001), with participants aged ≥65 years reporting the lowest satisfaction (86.79 ± 12.98), while younger age groups had higher scores. A similar pattern was found in the Eastern region, where age significantly influenced satisfaction (p<0.001), with participants aged ≤18 years reporting the highest scores (99.21 ± 1.02), while those aged 19–29 years had the lowest (86.23 ± 17.67). Gender differences were also significant in the Eastern region (p<0.001), with females reporting higher satisfaction (92.71 ± 10.29) than males (86.28 ± 17.6). In the Western region, satisfaction varied significantly across age groups (p<0.001), with older participants (≥65 years) reporting higher scores (93.51 ± 10.04) compared to younger groups. Gender differences were also noted (p=0.011), with females reporting higher satisfaction (90.92 ± 12.47) than males (88.34 ± 14.17).

In the Southern region, significant differences were observed across age groups (p=0.006), with participants aged 30–64 years reporting lower satisfaction (91.02 ± 14.24) compared to older and younger groups. Satisfaction also varied significantly across regions overall (p=0.025), with the Southern region reporting the highest scores (91.37 ± 14.18) and the Eastern region the lowest (88.60 ± 15.59). When comparing gender differences across all regions, females had significantly higher satisfaction than males (p=0.044), with an overall satisfaction score of 91.96 ± 12.15 compared to 88.91 ± 14.6 in males. Lastly, significant differences across age groups (p=0.002) and regions (p<0.001) were observed in the total sample, indicating that demographic factors, particularly gender and region, are statistically associated overall satisfaction scores, as shown in **Supplementary File C/Table 5**. Taken together, these subgroup findings show that demographic and regional factors are statistically associated with satisfaction scores, but the absolute differences in mean values are generally small and of limited clinical importance.

## 4 Discussion

This study provides a comprehensive assessment of patient satisfaction with dialysis services in Saudi Arabia, revealing generally high satisfaction levels with important variations across regions, facility types, demographic factors, and service domains. Overall satisfaction scored 89.84 ± 14.25, with the highest satisfaction observed in personal experience and the lowest in the dialysis domain. The generally positive satisfaction scores observed across all domains suggest that dialysis services in Saudi Arabia are largely meeting patient expectations. Several studies from Saudi Arabia reported similar high satisfaction score with dialysis centers (9, 10). When contextualized within the broader hemodialysis satisfaction literature, our scores appear to fall toward the upper end of the moderate–high satisfaction range described nationally and internationally. A multicenter Saudi study reported a mean overall dialysis satisfaction of 7.41 ± 2.75 on a 0–10 scale, indicating generally favorable but less uniformly high ratings than those seen in our national cohort (10). Studies from Egypt and other countries have similarly shown that most hemodialysis patients report being “satisfied” or “very satisfied” with dialysis care, with mean scores around the mid-points of 4- or 10-point scales and important deficits in specific aspects of care such as nutrition, information provision, or physician communication (20–22). Although these comparisons suggest that patient-reported satisfaction with dialysis services in Saudi Arabia is at least comparable to, and in some domains possibly higher than, that reported in other countries, differences in instruments, response scales, and scoring procedures preclude formal cross-study equivalence and these contrasts should therefore be interpreted descriptively rather than as direct statistical benchmarks. Moreover, most dialysis satisfaction studies, including those from Saudi Arabia, are cross-sectional snapshots rather than repeated surveys, so robust inferences about temporal trends in satisfaction, either nationally or internationally, are not currently possible and remain an important area for future research.

The personal experience domain had a numerically high mean score (91.39 ± 17.02), with 78.5% of patients expressing extreme satisfaction with staff teamwork; however, domain rankings should be viewed cautiously, as the scalar properties and cross-domain comparability of the scores have not been formally established. Patients’ experiences within a healthcare facility significantly shape their satisfaction, beginning with their first impressions of accessibility, location, and infrastructure (23). For dialysis patients, frequent visits make seamless access, adequate parking, and a calm environment essential for reducing stress and enhancing their treatment experience (24, 25). Additionally, factors such as cleanliness, safety, modern equipment, and comfortable amenities, including seating and entertainment options, play a vital role in improving patient comfort and fostering a positive care experience (26, 27). These environmental enhancements not only contribute to patient satisfaction but also have the potential to increase adherence to treatment regimens, enhance patient loyalty, and improve profit, especially in the private sector. A clean and safe environment can reduce anxiety and promote a sense of well-being, encouraging patients to consistently attend dialysis sessions and engage actively in their care.

The care domain also demonstrated high satisfaction, with 82.7% of patients feeling they were treated with respect and dignity. This domain was among those with the strongest correlations with overall satisfaction (r=0.905, p<0.001), suggesting that what patients experience in their day-to-day interactions with staff is closely aligned with their global judgments of dialysis care. In the Saudi sociocultural context, where family values, respect, and social harmony are emphasized, patients may particularly value healthcare providers who exhibit empathy, active listening, and cultural sensitivity, fostering greater trust and loyalty (28). Aligning with the results and interpretations of our study, prior research by Nemati et al. (29) underscored the significant impact of healthcare providers’ behavior, specifically doctors and nurses, on patient satisfaction levels (p < 0.001) (29). In a study conducted by Mansour et al. (30), it was revealed that patients demonstrated substantial satisfaction rates with nursing care (86.5%) and the quality of communication between patients and nurses (90.4%) (30). These findings also align with the results of Ferrans et al. (31), where patients indicated the highest satisfaction levels with aspects related to physician care, followed by nursing services and dialysis treatment (31). Moreover, a study conducted at Kenyatta National Hospital in Nairobi, Kenya, at a global level, demonstrated an overall satisfaction rate of 67.8% with nursing services; however, dissatisfaction was linked to an inadequate nurse-patient ratio and a limited number of dialysis machines (32). Collectively, these studies provide further support for the pivotal role of healthcare providers, particularly doctors and nurses, in influencing patient satisfaction within healthcare settings. However, these associations should be understood as strong but observational relationships, consistent with, but not definitive proof of, the idea that both the technical and interpersonal dimensions of care matter to patients.

Similarly, examining the level of satisfaction with the hemodialysis unit of Lahore General Hospital in Pakistan indicated that most patients (82.56%) were satisfied with the care received, except for the time spent with doctors, which supports the results of the current study (21). These findings highlight the importance of physicians and nursing care and resource availability in shaping patient satisfaction. In our study, we found that while 76.7% of patients were extremely satisfied with staff monitoring during dialysis sessions, only 61.6% expressed similar satisfaction with instructions on managing complications at home. This disparity suggests that patients may feel less confident and supported in self-managing their care outside the clinical setting. Contributing factors include limited patient education, lack of real-time assistance, and insufficient training resources for home dialysis. Addressing these issues through enhanced education, personalized support, and improved resource allocation can help improve patient satisfaction with home dialysis care.

In contrast, the dialysis domain received the lowest satisfaction rating (88.45 ± 18.65), among the six domains assessed. While this highlights opportunities for improvement, particularly in patient education on managing complications at home, it is important to note that the absolute score remains high, suggesting a potential ceiling effect in patient satisfaction measurement. Within our distribution-based MCID framework, this gap between the dialysis domain and the highest-scoring domains represents a small effect size, indicating that this domain is statistically lower but only modestly different in perceived care quality. However, this context indicates that even “lower” domains reflect strong overall ratings, which may limit the apparent scope for measurable improvement. Particular concern was the relatively lower satisfaction (61.6%) with instructions on managing dialysis complications at home, highlighting a potential gap in patient education and self-management support. This finding aligns with both global and regional studies, which have similarly reported patient dissatisfaction with education and instructions related to their disease and dialysis interventions (33–35). Clear and comprehensive instructions from healthcare staff significantly impact hemodialysis patient satisfaction by empowering them with knowledge about their condition, treatment options, and long-term outcomes (27, 36). Additionally, implementing a team-based care model that engages various healthcare providers, including health educators, health promotion specialists, and health coaches, can distribute tasks among nurses and doctors, enhance patient education, improve tracking and monitoring, and ultimately increase patient satisfaction and experience. Finally, the quality of the facility is closely tied to the effectiveness of information delivery, further influencing patient perceptions of care (37).

The significant regional variations in satisfaction scores deserve particular attention. The Southern region consistently demonstrated the highest satisfaction across most domains, while the Eastern region reported the lowest overall satisfaction. The statistically significant yet clinically small regional variations in dialysis satisfaction observed in our study may reflect broader patterns of healthcare disparity across Saudi Arabia that have been consistently observed in multiple healthcare domains (38–41). These variations likely stem from a complex interplay of factors, including resource allocation, cultural differences, population demographics, and implementation of quality initiatives (41, 42). Addressing these disparities requires multifaceted approaches that combine targeted resource allocation, transfer of best practices, standardized quality assurance, and regular monitoring of patient experiences. Future initiatives should also engage patients and communities to listen to their voices, ensuring that interventions are patient-centered and responsive to their needs.

The higher satisfaction reported in private facilities (90.41 ± 13.31) compared to governmental hospitals (88.57 ± 16.08) aligns with findings from other Saudi Arabian studies examining healthcare satisfaction more broadly. Although this difference reached statistical significance in our large sample, the 2-point gap in mean overall satisfaction corresponds to a small effect size within our distribution-based MCID framework and is unlikely to represent a major difference in perceived care quality. Alodhialah et al. (43) found that private hospitals in Saudi Arabia consistently outperformed public facilities in patient satisfaction metrics, particularly regarding waiting times and facility amenities (43). The relatively higher performance of private healthcare facilities in Saudi Arabia may be partly related to greater investment in infrastructure, technology, and staff training, and to competitive market dynamics that prioritize patient satisfaction. However, unmeasured factors, such as differences in patient demographics, case mix, or expectations, may also contribute to these differences and should be considered when interpreting these findings (44, 45). To improve public healthcare quality, policymakers may consider initiatives such as enhanced staff training, lower patient-to-provider ratios, and adopting best practices from the private sector (11). The slight, non-significant difference between the two private providers suggests relatively standardized care quality across private dialysis providers, which contrasts with greater variability observed in governmental facilities in previous studies. Future research should explore the specific organizational practices and patient-centered strategies adopted by private providers that could be effectively implemented within governmental settings to reduce variability and enhance overall dialysis care quality.

The demographic variations in satisfaction scores provide valuable insights for targeted service improvements. The markedly higher satisfaction among the youngest patients (≤18 years) compared to young adults (19–29 years) raises essential questions about transition care, which is the process of moving from pediatric to adult dialysis services, and age-appropriate service delivery. The absolute differences between the ≤18-year group and older age groups were approximately 4–7 points, corresponding to small-to-moderate effect sizes that likely have some clinical relevance, particularly given the developmental importance of autonomy and social participation in adolescence and young adulthood. The lower satisfaction among young adults may reflect the particular challenges they face in balancing dialysis requirements with education, career development, and social life aspirations. Pediatric (≤18 years) dialysis services typically provide more personalized care with higher staff-to-patient ratios and greater parental involvement, potentially creating a more supportive environment. Moreover, pediatric patients (≤18 years) often benefit from specialized psychosocial support services tailored to their developmental needs. Finally, parental advocacy may contribute to higher ratings among pediatric patients, as parents typically complete satisfaction surveys for minors and may report more positively than young adults (19–29 years) completing their own assessments (46). On the other hand, young adults simultaneously navigate crucial developmental milestones, including higher education, career establishment, identity formation, and relationship building, all significantly complicated by the demanding nature of dialysis treatment schedules, which affect their quality of life. The transition from family-centered pediatric care to adult-oriented services frequently lacks structured transition programs, creating a service gap (47). Additionally, the rigid treatment regimens pose greater perceived limitations on independence and social integration for young adults who are developmentally driven to establish autonomy (48).

The observed gender differences in satisfaction scores toward dialysis in this study are consistent with findings from other studies investigating satisfaction with general healthcare services in the MENA region (49, 50). Al Eissa et al. (10) observed similar gender differences in a study of primary healthcare satisfaction in Saudi Arabia, suggesting that gender-specific communication styles and expectations may influence satisfaction ratings (10). Cultural factors, such as patients’ preferences for receiving care from healthcare providers of the same gender (49), may help explain these differences in satisfaction and warrant further investigation (51). However, at the same time, the absolute between-group differences in mean scores were small, typically in the range of 2–3 points on a 0–100 scale, and therefore modest relative to the observed standard deviations (approximately 14–17 points). In the absence of an empirically derived MCID for this survey, these values correspond to effect sizes below the commonly used 0.5 standard deviation threshold for a moderate, potentially clinically important difference, and should be interpreted as statistically significant associations of uncertain clinical relevance from the patient perspective.

### 4.1 Clinical implications

Our findings have several important clinical implications for dialysis service delivery. The strong correlation between the care domain and overall satisfaction (r=0.905) suggests that investments in staff training focused on communication skills, empathy, and patient-centered care approaches may yield significant improvements in patient satisfaction. This aligns with the study of Kshirsagar et al. (52), who highlighted the significant impact of patient-centered care training programs for dialysis staff on patients’ satisfaction (52). However, given our large sample size, even small differences in mean scores reached statistical significance; therefore, quality-improvement initiatives should prioritize changes that are large enough to shift domain scores by at least a moderate effect, which are more likely to be perceived by patients as clinically meaningful improvements in their experience.

The relatively lower satisfaction with instructions on managing dialysis complications at home (61.6% extremely satisfied) highlights an urgent need for enhanced patient education and self-management support, potentially through the integration of digital health technologies such as mobile applications, tele-education platforms, and interactive educational tools. Structured education programs incorporating teach-back methods, multimedia resources, and regular reinforcement could address this gap. Nemati et al. (29) proposed that prioritizing patient education and effective communication can greatly enhance patient satisfaction and contribute to a better quality of life for individuals undergoing dialysis (29).

The pharmacy domain findings, particularly regarding medication availability (65.8% extremely satisfied), suggest supply chain and inventory management issues that require attention. Regular medication reconciliation, improved coordination between dialysis centers and pharmacies, and investment in pharmacy infrastructure could enhance this aspect of care (53).

### 4.2 Strengths and limitations

This study has several notable strengths that enhance its contribution to the literature. The large sample size (5,472 participants) from multiple regions provides robust statistical power and valuable insights across Saudi Arabia. The inclusion of both governmental and private facilities, including the two major private providers (Diaverum and DaVita), offers valuable comparative insights rarely available in previous research. The use of the validated Press Ganey instrument, administered by an independent third party, enhances the reliability of the findings and reduces potential bias in data collection. The comprehensive assessment across six domains provides a nuanced understanding of patient satisfaction beyond single composite measures.

Despite these strengths, several limitations must be acknowledged. The cross-sectional design prevents the establishment of causal relationships or trends over time. The self-reported nature of the data introduces recall bias and potential social desirability bias. The study is subject to potential selection and non-response bias. As we utilized a de-identified secondary dataset extracted from the national MOH patient experience program, information on the total number of active dialysis patients, the number of eligible patients invited to participate, and the number of non-responders was unavailable, and the overall response rate could not be calculated. This prevents a formal analysis of non-response bias and requires caution when generalizing the findings to the entire dialysis patient population in Saudi Arabia. Furthermore, in the absence of an anchor-based MCID for this satisfaction instrument, we relied on a distribution-based interpretation of effect sizes. Although this is a widely used approach for patient-reported outcomes, our classification of differences as ‘clinically meaningful’ versus ‘statistically significant but small’ should be interpreted with caution until validated against patient-anchored thresholds in the dialysis population. Additionally, limited clinical information about participants’ comorbidities, dialysis vintage, or treatment modality prevents analysis of how these factors might influence satisfaction. Our sample had more patients from private facilities, which may limit the generalizability of public–private satisfaction comparisons. The small pediatric stratum also reduces the precision and robustness of age-specific conclusions. Additionally, the questionnaire, while comprehensive, may not capture culture-specific aspects of satisfaction relevant to the Saudi context.

### 4.3 Future research directions

This study suggests several promising avenues for future research. Longitudinal studies tracking satisfaction over time would provide valuable insights into how service changes and healthcare reforms impact patient experiences. Mixed methods approach incorporating qualitative interviews or focus groups would enhance understanding of the factors underlying satisfaction ratings, particularly for domains with lower scores. Intervention studies testing specific strategies to address identified weaknesses, such as enhanced patient education programs or pharmacy service improvements, would generate practical evidence for quality improvement. Exploration of the relationship between satisfaction metrics and clinical outcomes, including treatment adherence, hospitalization rates, and mortality, would strengthen the case for investing in satisfaction-enhancing initiatives. Studies specifically examining the experiences of young adult patients (19–29 years) and male patients could identify targeted approaches to address their relatively lower satisfaction. Finally, economic analyses evaluating the cost-effectiveness of various satisfaction improvement strategies would provide valuable information for resource allocation decisions.

## 5 Conclusions

This comprehensive assessment of patient satisfaction with dialysis services across Saudi Arabia reveals generally high satisfaction levels with significant variations across regions, facility types, and demographic factors. The strong correlation between interpersonal aspects of care and overall satisfaction emphasizes that technical excellence alone is insufficient; compassionate, respectful delivery of care is equally crucial. The findings provide actionable insights for policymakers and healthcare providers to enhance service quality, particularly regarding regional equity, patient education, and pharmacy services. The observed disparities between governmental and private facilities, as well as regional variations, highlight the need for standardized quality improvement initiatives and targeted resource allocation. The demographic differences in satisfaction suggest opportunities for personalized approaches to care delivery that account for age and gender-specific needs and expectations.

## Data Availability

The original contributions presented in the study are included in the article/**Supplementary Material**. Further inquiries can be directed to the corresponding author.
