# Supplementary material for: Patient satisfaction with dialysis services provided across different providers in Saudi Arabia
Source: Front Nephrol. 2026 Jan 12;5:1691773. doi: 10.3389/fneph.2025.1691773 (PMC12832406; doi:10.3389/fneph.2025.1691773)
Supplement: Supplementary file 2 [file SupplementaryFile2.docx]

**Supplementary Table 1: Comparison for the Overall Rating of Care received across Regions, Hospital Types, Age Groups, and Gender**

| **Parameter** | **n** | **Mean Score (95% CI)** | **Difference (95% CI)** | **p-value** |
| --- | --- | --- | --- | --- |
| **Regions** |  |  |  | <0.001 |
| Central | 1341 | 90.10 (89.37, 90.82) | Ref | - |
| Eastern | 735 | 88.60 (87.48, 89.73) | -1.49 (-2.77, -0.21) | 0.022 |
| Northern | 465 | 89.13 (87.75, 90.50) | -0.97 (-2.47, 0.53) | 0.207 |
| Southern | 1258 | 91.37 (90.59, 92.16) | 1.28 (0.18, -2.37) | 0.022 |
| Western | 1662 | 89.18 (88.51, 89.85) | -0.91 (-1.94, 0.11) | 0.080 |
| **Hospital Type** |  |  |  |  |
| Governmental | 1717 | 88.57 (87.81, 89.33) | Ref | - |
| Private | 3744 | 90.41 (89.98, 90.83) | 1.83 (1.02, 2.65) | <0.001 |
| **Private Hospital Type** |  |  |  |  |
| Diaverum | 1744 | 90.70 (90.11, 91.30) | Ref | - |
| Davita | 2000 | 90.15 (89.54, 90.75) | -0.55 (-1.41, 0.29) | 0.203 |
| **Age Group** |  |  |  | 0.075 |
| <=18 | 56 | 93.80 (90.74, 96.86) | Ref | - |
| 19-29 | 364 | 89.18 (87.67, 90.69) | -4.62 (-8.54, -0.70) | 0.021 |
| 30-64 | 3538 | 89.90 (89.43, 90.36) | -3.90 (-7.58, -0.23) | 0.037 |
| >=65 | 1118 | 90.47 (89.70, 91.25) | -3.33 (-7.06, 0.41) | 0.081 |
| **Gender** |  |  |  |  |
| Male | 3099 | 88.91 (88.39, 89.42) | Ref | - |
| Female | 1972 | 91.96 (91.42, 92.49) | 3.05 (2.31, 3.79) | <0.001 |

P-Value: The probability of observing the data, assuming no true difference exists. A p-value <0.05 indicates statistical significance. Statistical Tests: Differences will be analyzed using t-tests for two-group comparisons and ANOVA for multi-group comparisons. Significance Threshold: Results are considered statistically significant at p < 0.05.

**Supplementary Table 2: Post-hoc analysis comparing satisfaction scores across regions**

| **(I) Region** | **(J) Region** | **Mean Difference (I-J)** | **Slandered Error** | **p-value** | **95% Confidence Interval** | |
| --- | --- | --- | --- | --- | --- | --- |
|  |  |  |  |  | **Lower Bound** | **Upper Bound** |
| **Central** | Eastern | 1.49 | 0.65 | 0.022 | 0.21 | 2.77 |
|  | Northern | 0.97 | 0.77 | 0.207 | -0.53 | 2.47 |
|  | Western | 0.91 | 0.52 | 0.080 | -0.11 | 1.94 |
|  | Southern | -1.28 | 0.56 | 0.022 | -2.37 | -0.18 |
| **Eastern** | Central | -1.49 | 0.65 | 0.022 | -2.77 | -0.21 |
|  | Northern | -0.52 | 0.84 | 0.535 | -2.18 | 1.13 |
|  | Western | -0.58 | 0.63 | 0.360 | -1.81 | 0.66 |
|  | Southern | -2.77 | 0.66 | <0.001 | -4.06 | -1.47 |
| **Northern** | Central | -0.97 | 0.77 | 0.207 | -2.47 | 0.53 |
|  | Eastern | 0.52 | 0.84 | 0.535 | -1.13 | 2.18 |
|  | Western | -0.05 | 0.75 | 0.943 | -1.52 | 1.41 |
|  | Southern | -2.25 | 0.77 | 0.004 | -3.76 | -0.73 |
| **Western** | Central | -0.91 | 0.52 | 0.080 | -1.94 | 0.11 |
|  | Eastern | 0.58 | 0.63 | 0.360 | -0.66 | 1.81 |
|  | Northern | 0.05 | 0.75 | 0.943 | -1.41 | 1.52 |
|  | Southern | -2.19 | 0.53 | <0.001 | -3.24 | -1.15 |
| **Southern** | Central | 1.28 | 0.56 | 0.022 | 0.18 | 2.37 |
|  | Eastern | 2.77 | 0.66 | <0.001 | 1.47 | 4.06 |
|  | Northern | 2.25 | 0.77 | 0.004 | 0.73 | 3.76 |
|  | Western | 2.19 | 0.53 | <0.001 | 1.15 | 3.24 |

**Supplementary Table 3: Post-hoc analysis comparing satisfaction scores across age groups**

| **(I) Age** | **(J) Age** | **Mean Difference (I-J)** | **Slandered Error** | **p-value** | **95% Confidence Interval** | |
| --- | --- | --- | --- | --- | --- | --- |
|  |  |  |  |  | **Lower Bound** | **Upper Bound** |
| <=18 | 19-29 | 4.62 | 2 | 0.021 | 0.70 | 8.54 |
|  | 30-64 | 3.90 | 1.87 | 0.037 | 0.22 | 7.58 |
|  | >=65 | 3.33 | 1.91 | 0.081 | -0.41 | 7.06 |
| 19-29 | <=18 | -4.62 | 2 | 0.021 | -8.54 | -0.70 |
|  | 30-64 | -0.72 | 0.77 | 0.348 | -2.22 | 0.78 |
|  | >=65 | -1.29 | 0.84 | 0.124 | -2.94 | 0.35 |
| 30-64 | <=18 | -3.9 | 1.87 | 0.037 | -7.58 | -0.23 |
|  | 19-29 | 0.71 | 0.77 | 0.348 | -0.78 | 2.22 |
|  | >=65 | -0.57 | 0.48 | 0.230 | -1.51 | 0.36 |
| >=65 | <=18 | -3.33 | 1.9 | 0.081 | -7.06 | 0.41 |
|  | 19-29 | 1.29 | 0.83 | 0.124 | -0.35 | 2.94 |
|  | 30-64 | 0.57 | 0.48 | 0.230 | -0.36 | 1.51 |

**Supplementary Table 4: Correlation analysis**

| **Domain** | **Correlation Coefficient (r)** | **p-value** |
| --- | --- | --- |
| **Correlation between Overall Satisfaction and Scores of Domains** | | |
| Registration | 0.791 | <0.001 |
| Care | 0.905 | <0.001 |
| Dialysis | 0.864 | <0.001 |
| Pharmacy | 0.721 | <0.001 |
| Personal Issues | 0.833 | <0.001 |
| Personal Experience | 0.885 | <0.001 |
| **Correlation between Highest Correlated Domain (Care) and Scores of questions inside that Domain** | | |
| Medical staff's explanation of the dialysis procedure | 0.818 | <0.001 |
| Medical staff provided opportunity to ask questions | 0.812 | <0.001 |
| Your trust in the skill of the medical staff | 0.828 | <0.001 |
| Staff's concern for your comfort | 0.816 | <0.001 |
| Staff treated you with respect and dignity | 0.758 | <0.001 |
| Response to concerns/complaints made during your visit | 0.809 | <0.001 |
| Correlation between Lowest Correlated Domain (Pharmacy) and Scores of questions inside that Domain | | |
| Explanations provided by pharmacist about prescription(s) | 0.822 | <0.001 |
| Availability of prescribed medications | 0.915 | <0.001 |

**Supplementary Table 5: Subgroup comparison showing the effect of demographics on overall satisfaction**

| **Region** | **Gender** | **Age Group** | | | | **Total**  **n (Mean ± SD)** | **p-value*** |
| --- | --- | --- | --- | --- | --- | --- | --- |
|  |  | **<=18**  **n (Mean ± SD)** | **19-29**  **n (Mean ± SD)** | **30-64**  **n (Mean ± SD)** | **>=65**  **n (Mean ± SD)** |  |  |
| Central | Male | 15 92.84 ± 10.36 | 43 88.66 ± 17.1 | 483 90.31 ± 12.66 | 129 86.79 ± 12.98 | 670 89.58 ± 13.05 | 0.04 |
|  | Female | 6 94.07 ± 7.62 | 33 90.18 ± 12.92 | 305 91.93 ± 12.78 | 99 93.37 ± 9.68 | 444 92.06 ± 12.27 | 0.487 |
|  | Total | 21 93.19 ± 9.48 | 76 89.32 ± 15.35 | 789 90.95 ± 12.72 | 228 89.65 ± 12.09 | 1341 90.1 ± 13.48 | 0.267 |
|  | P-value$ | 0.769 | 0.659 | 0.082 | <0.001 | 0.001 |  |
| Eastern | Male | 4 99.65 ± 0.69 | 27 82.85 ± 21.48 | 359 86.6 ± 17.7 | 49 85.02 ± 15 | 443 86.28 ± 17.6 | <0.001 |
|  | Female | 3 98.61 ± 1.2 | 19 91.04 ± 8.5 | 169 93.27 ± 10.81 | 57 91.37 ± 9.8 | 255 92.71 ± 10.29 | <0.001 |
|  | Total | 7 99.21 ± 1.02 | 46 86.23 ± 17.67 | 528 88.73 ± 16.12 | 106 88.43 ± 12.81 | 735 88.6 ± 15.59 | <0.001 |
|  | P-value$ | 0.272 | 0.082 | <0.001 | 0.013 | <0.001 |  |
| Northern | Male | 8 92.19 ± 20.85 | 31 90.73 ± 14.97 | 140 87.53 ± 14.9 | 41 90.51 ± 10.61 | 222 88.7 ± 14.42 | 0.46 |
|  | Female | 4 92.08 ± 13.2 | 25 91.23 ± 13 | 93 91.97 ± 11.06 | 34 86.59 ± 20.04 | 160 90.88 ± 13.76 | 0.585 |
|  | Total | 12 92.15 ± 18 | 62 90.33 ± 13.74 | 245 89.09 ± 13.93 | 84 87.86 ± 15.76 | 465 89.13 ± 15.08 | 0.724 |
|  | P-value$ | 0.992 | 0.893 | 0.01 | 0.31 | 0.135 |  |
| Western | Male | 1 93.75 ± 0 | 70 88.31 ± 13.7 | 764 88.58 ± 14.2 | 188 87.39 ± 14.29 | 1024 88.34 ± 14.17 | - |
|  | Female | 3 96.64 ± 3.15 | 45 87.81 ± 15.29 | 421 90.27 ± 12.84 | 151 93.51 ± 10.04 | 621 90.92 ± 12.47 | 0.011 |
|  | Total | 4 95.92 ± 2.95 | 115 88.11 ± 14.28 | 1188 89.14 ± 13.86 | 342 89.65 ± 14.36 | 1662 89.18 ± 13.99 | 0.005 |
|  | P-value$ | - | 0.859 | 0.037 | <0.001 | <0.001 |  |
| Southern | Male | 4 89.67 ± 16.37 | 42 91.33 ± 13.03 | 474 89.93 ± 15.26 | 217 92.45 ± 12.29 | 740 90.72 ± 14.35 | 0.225 |
|  | Female | 8 94.14 ± 9.6 | 23 92.9 ± 11.75 | 314 92.65 ± 12.4 | 140 93.86 ± 11.12 | 492 93.13 ± 11.89 | 0.775 |
|  | Total | 12 92.65 ± 11.69 | 65 91.89 ± 12.52 | 788 91.02 ± 14.24 | 358 93 ± 11.83 | 1258 91.37 ± 14.18 | 0.127 |
|  | P-value$ | 0.640 | 0.624 | 0.006 | 0.263 | 0.001 |  |
| Males | | 32 93.16 ± 13.41 | 213 88.64 ± 15.7 | 2220 88.86 ± 14.83 | 624 89.04 ± 13.43 | 3099 88.91 ± 14.6 | 0.349 |
| Females | | 24 94.65 ± 8.26 | 145 90.17 ± 13.04 | 1302 91.74 ± 12.38 | 481 92.84 ± 11.35 | 1972 91.96 ± 12.15 | 0.044 |
| Total | | 56 93.8 ± 11.42 | 364 89.18 ± 14.62 | 3538 89.9 ± 14.1 | 1118 90.47 ± 13.21 | 5472 89.84 ± 14.25 | 0.034 |
| P-value Male# | | - | 0.406 | 0.004 | <0.001 | <0.001 |  |
| P-value Female# | | 0.466 | 0.656 | 0.034 | 0.187 | 0.024 |  |
| P-value Total# | | 0.025 | 0.274 | 0.002 | <0.001 | <0.001 |  |

*Comparison between age groups using Welch ANOVA

$Comparison between genders using independent t-test

#Comparison between regions using Welch ANOVA

-Comparison is not possible
