# Supplementary material for: Patient satisfaction with dialysis services provided across different providers in Saudi Arabia
Source: Front Nephrol. 2026 Jan 12;5:1691773. doi: 10.3389/fneph.2025.1691773 (PMC12832406; doi:10.3389/fneph.2025.1691773)
Supplement: Supplementary file 3 [file SupplementaryFile3.docx]

**Supplementary File B. Psychometric properties of the Press Ganey dialysis patient experience survey**

The dialysis patient experience survey used in this study is a 22-item, dialysis-specific adaptation of the Press Ganey patient experience instruments. Items are grouped into six domains reflecting key aspects of dialysis care: registration (4 items), care (6 items), dialysis process (3 items), pharmacy services (3 items), personal interactions (2 items), and overall assessment (4 items). Each item is rated on a 5-point Likert scale from 1 (“not satisfied at all”) to 5 (“extremely satisfied”). Item scores are linearly transformed to a 0–100 scale and averaged within each domain to generate domain scores; an overall satisfaction score is computed as the mean of all applicable items, with higher scores indicating greater satisfaction.

At an international level, Press Ganey surveys have been shown to exhibit a clear multidimensional structure, high internal consistency reliability across domains, and evidence of construct, convergent, divergent, and predictive validity in large samples of patients receiving outpatient care. In a large US outpatient cohort, Presson et al. demonstrated acceptable internal consistency (Cronbach’s α 0.79–0.96), a stable multi-domain factor structure, and evidence of convergent and divergent validity for the Outpatient Medical Practice Survey (1). Cross-cultural validations have confirmed good model fit and high reliability for translated versions, including an Arabic adaptation used in hospitals in Saudi Arabia and the UAE, which showed strong reliability and construct, convergent, divergent and predictive validity (2). Vendor technical reports and independent psychometric studies support the use of these instruments as measures of patient experience in routine clinical practice. In the Saudi context, the dialysis survey was translated into Arabic and culturally adapted by the Ministry of Health in collaboration with Press Ganey following World Health Organization guidelines for instrument translation and adaptation, which include forward translation, expert panel review, back-translation, and pre-testing. To our knowledge, an empirically derived minimal clinically important difference (MCID) specific to this dialysis survey has not yet been established; therefore, in the present study we interpret between-group differences descriptively and in relation to the observed variability in scores rather than as formally validated clinical thresholds.

References

1. Presson AP, Zhang C, Abtahi AM, Kean J, Hung M, Tyser AR. Psychometric properties of the Press Ganey® Outpatient Medical Practice Survey. Health Qual Life Outcomes. 2017 Feb;15(1).

2. Malott DL, Fulton BR, Rigamonti D, Myers S. Psychometric Testing of a Measure of Patient Experience in Saudi Arabia and the United Arab Emirates. J Surv Stat Methodol [Internet]. 2017 Sep 1;5(3):398–408. Available from: https://doi.org/10.1093/jssam/smx008
